# Influence of Music on the Behaviors of Crowd in Urban Open Public Spaces

**DOI:** 10.3389/fpsyg.2018.00596

**Published:** 2018-04-27

**Authors:** Qi Meng, Tingting Zhao, Jian Kang

**Affiliations:** ^1^Heilongjiang Cold Region Architectural Science Key Laboratory, School of Architecture, Harbin Institute of Technology, Harbin, China; ^2^UCL Institute for Environmental Design and Engineering, University College London, London, United Kingdom

**Keywords:** music, crowd behavior, movement, soundscape, urban open space

## Abstract

Sound environment plays an important role in urban open spaces, yet studies on the effects of perception of the sound environment on crowd behaviors have been limited. The aim of this study, therefore, is to explore how music, which is considered an important soundscape element, affects crowd behaviors in urban open spaces. On-site observations were performed at a 100 m × 70 m urban leisure square in Harbin, China. Typical music was used to study the effects of perception of the sound environment on crowd behaviors; then, these behaviors were classified into movement (passing by and walking around) and non-movement behaviors (sitting). The results show that the path of passing by in an urban leisure square with music was more centralized than without music. Without music, 8.3% of people passing by walked near the edge of the square, whereas with music, this percentage was zero. In terms of the speed of passing by behavior, no significant difference was observed with the presence or absence of background music. Regarding the effect of music on walking around behavior in the square, the mean area and perimeter when background music was played were smaller than without background music. The mean speed of those exhibiting walking around behavior with background music in the square was 0.296 m/s slower than when no background music was played. For those exhibiting sitting behavior, when background music was not present, crowd density showed no variation based on the distance from the sound source. When music was present, it was observed that as the distance from the sound source increased, crowd density of those sitting behavior decreased accordingly.

## Introduction

The phrase “urban open space” can describe many types of open areas (Marcus and Francis, 1998). One definition holds that, as the counterpart of development, urban open space is a natural and cultural resource, synonymous with neither unused land nor park and recreation areas. Another definition is that open space is land and/or water area with its surface open to the sky which has been consciously acquired or publicly regulated to serve conservation and urban shaping functions in addition to providing recreational opportunities (Myers, 1975; Thompson, 2002). In modern cities, the benefits that urban open space provides to citizens can be separated into three basic categories: recreation, ecology, and esthetic value (Brander and Koetse, 2011). Sound quality is considered to be a key part of the ecological/sustainable development of urban open spaces (Zhang et al., 2006). However, the sound environment of urban open spaces is often not satisfactory because of a lack of consideration for human behavior during the planning and managing of the spaces (Meng and Kang, 2016). Therefore, research on the effect of perception of the sound environment on crowd behavior will be of importance to landscape research in this field. According to the International Standards Organization, a “soundscape” is defined as an acoustic environment as perceived/experienced in context (ISO 12913-1, 2014). Behavior comes into play in soundscape assessment in that the activities and behaviors of surrounding people form a key facet of context.

Individual behaviors generally refer to the attitude or performance of a person in certain situations; their actions can be largely random, subject to the effect of the environment (Jia, 2012). In contrast, crowd behaviors refer to the attitude or performance of a crowd in an environment; they can be composed of certain regularities, subject to the effect of the environment (Yuan and Tan, 2011; Xie et al., 2013). Thus, instead of individual behaviors, crowd behaviors are usually examined in studies on urban open spaces (Marušić, 2011; Lepore et al., 2016; Meng and Kang, 2016). Lewin et al. (1936) presented the following formula, which indicates the interaction between an individual and his/her environment: B = f (P, E), in which B represents behavior; P represents persons, including individuals and groups; and E represents the environment in which those persons live. Based on this formula, both users’ social characteristics and local environment must be considered in human behavior studies. Previous studies pointed out that recreational behavior can be affected by users’ cultural background, age, and different local areas (Floyd, 1998; Payne et al., 2002; Guéguen et al., 2008).

Many different aspects of crowd behavior can be examined to draw conclusions. These can include characteristics of behaviors, such as movement and action (Wang, 2014); characteristics of movement, for example, characterized as movement or non-movement, with the former including passing by and walking around and the latter including sitting (Chen, 2009); and characteristics of actions, such as sitting, standing, watching, and loitering (Lepore et al., 2016). The number of participants is also an important factor, that is, whether the behavior involves one person, two people, or multiple people (Jia, 2012); the intrinsic properties of such behaviors can also be examined, such as whether they are necessary, spontaneous, or social (Gehl, 1987). The frequency and location of the behavior are also important, such as whether it is neighborhood or urban behavior (Chen, 2009). Additionally, factors such as crowd behavior in the sound environment, participation behavior, tendency behavior, avoiding behavior, and other behaviors which are not affected by the environment are all significant angles that reveal crucial information regarding crowd behaviors (Jia, 2012).

The sound environment can affect human perception, and human perception can influence crowd behavior in both indoor and outdoor spaces. For example, previous studies have demonstrated that environmental music affects the pace of shopping and amount of time spent in shopping malls (Milliman, 1982). Other studies have also shown that eating and talking behavior can be affected by background music in dining spaces (Fiegel et al., 2014; Meng et al., 2017a). In urban open spaces, studies have found that people who pass by will stop to stand and watch music-related activities, whereas the amount of exercising behavior will be changed a little by music-related activities (Meng and Kang, 2016). Another study indicated that the presence of music can prolong the duration of stay in a tunnel when compared with silence, and classical music caused the longest duration of stay (Aletta et al., 2016). It has also been found that in the case of sound stimulation in the audio–visual environment of the countryside, study participants’ gazing range was demonstrated to be significantly more dispersed than when no sound stimulation was present (Ren and Kang, 2015). Previous studies have mainly focused on the effect of the sound environment on one’s action (Zakariya et al., 2014; Aletta et al., 2016; Lepore et al., 2016). However, studies on the effects of certain typical sound sources on crowd behaviors classified as movement or non-movement have been limited.

Musical sound is a common sound source in urban open spaces (North et al., 2004; Styns et al., 2007). Studies have shown that when people listen to music, their emotions fluctuate, and the effect is to change their behavior (Orr et al., 1998). Studies have shown that different languages, tempos, tones, and sound levels of music can cause different effects on emotions, mental activities, and physical reactions. Overall, languages and tempos are the two most important factors (Sakharov et al., 2005; Carpentier and Potter, 2007). Other studies have found that fast music is associated with more activation than slow music (Gomez and Danuser, 2004; Natarajan et al., 2004). For example, a study researching participants with headphones found that fast music increases walking speed, while slow music causes slower walking speeds (Franěk et al., 2014).

The cited studies indicate that the sound environment can affect crowd behaviors; building on this finding, the present research focuses on the effects of music, an important soundscape element, on specific crowd behaviors, classified as movement, including passing by and walking around, or non-movement, including sitting. Previous studies indicate that path and speed are significant characteristics that describe movement behavior, while crowd density is important in describing non-movement behavior (Ye et al., 2012; Lavia et al., 2016). Therefore, the aims of this study are to find out: (1) whether music can change the path or speed of passing by or walking around behavior; we hypothesize that the speed of passing by or walking around behavior will increase with music, the path of passing by behavior will shift closer to the music, and the area or perimeter of walking around behavior will decrease with the music, since some previous studies have pointed out that music-related activities can increase the speed of passing by or walking around behavior in some urban open spaces and (2) whether music can decrease or increase sitting behaviors in urban open spaces; we hypothesize that the sitting behavior will increase with decreasing distance of music, since eating and talking behaviors can be affected by music. An urban leisure square was chosen as the case site, and music was chosen. In addition, three typical behaviors were selected for further analysis at the case site, and on-site observations were used for data collection. To achieve the aim of the study, several different approaches were explored. First, this study examined the effect of music on the path and speed of passing by behavior. Second, it determined the effect of music on the path and speed of walking around behavior. Third, it observed the effect of music on the location of sitting behavior.

## Materials and Methods

### Survey Site

**FIGURE 1:**
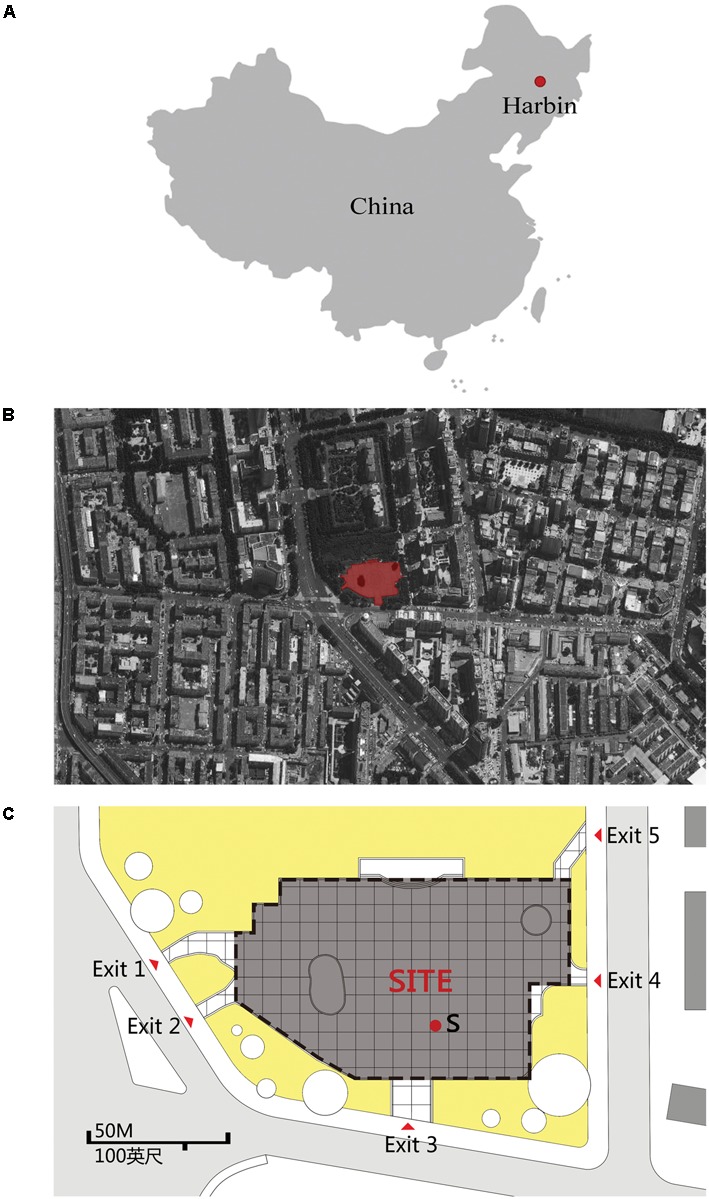
The map of LANDSCAPE square and the case site: **(A)** location of LANDSCAPE square, **(B)** map of LANDSCAPE square, and **(C)** the survey site.

Previous studies have indicated that an urban street is a kind of linear space, where people have little choice but for their paths to be confined to the pavement by buildings or motor vehicles (Hwang et al., 2011). In contrast, an urban square is an “areal” type of space, where people are free to choose their direction and path of travel (Marcus and Francis, 1998; Zakariya et al., 2014). In this study, a typical urban leisure square named “LANDSCAPE” square, located in Harbin, in Northeast China, was selected as the case site. Maps of the square and survey site can be found in **Figure 1**.

This site was chosen for the following three reasons. First, it is located at the crossing of Changjiang Road and Hongxiang Road; roads are present on three sides of the square, and vegetation is found on the fourth side. The square’s surrounding environment (determining its scale and format) resembles that of squares encountered in Europe and Japan (Ashihara, 1985; Whitlock, 2004), and thus has typicality. Next, the urban leisure square is nearly 100 m long and 70 m wide and covers an area of about 7000 m^2^, which is typical for modern cities (Dai, 2014). Finally, the square has various sports facilities and lush vegetation; thus, a large number of local residents visit the square and it is an important urban open space in this area for people to relax and interact. Overall, the square provides convenient conditions and the opportunity to gather a large number of samples to study crowd movement behaviors as well as non-movement behaviors.

Studies indicate that environmental changes, such as changes in temperature and humidity, influence subjective acoustic perception (Thwaites et al., 2005; dela Fuente de Val et al., 2006). To avoid the effects of these environmental factors, measurements were performed on workdays in May and September 2017. The mean monthly temperatures (18–26°C) and the relative humidities of these months are approximately the same. Other studies indicate that illuminance, which may also affect the perception or behaviors of users, changes with the time of day (Liu et al., 2013; Meng et al., 2017b). Thus, in the present study, measurements were performed between 9:00–11:00 a.m. and 14:00–16:00 p.m. daily on workdays to avoid the effects of time of day on the light environment (Meng et al., 2017a).

### Sound Source

In previous music studies, musical sound can be classified by sound level, tempo, genre, context, familiarity, and so on (Husain et al., 2002; Sakharov et al., 2005; Kang, 2017). Based on the method used in the experiment by Lavia et al. (2016), the music excerpts were designed to be “inclusive,” “non-aversive,” and sound good in a highly reverberant environment. In this study, a typically familiar pop song with lyrics, named “Free to Fly,” was selected as the stimulus for intervention in the acoustic environment of the square; the tempo of this song is 120 bpm.

A loudspeaker was used as the sound source; its location is shown in **Figure 1C** (S means sound source). The loudspeaker location was chosen for the following three reasons. First, the music played by the loudspeaker can be clearly heard at any point in the square. Second, the distance between the loudspeaker and the walls and other major reflective surfaces was ensured to be at least 20 m (Zahorik, 2002). Finally, to avoid any influence caused by the visual presence of the loudspeaker, it was placed near the water feature fence to avoid identification. During the experiment, the musical excerpt and silence were reproduced cyclically (Husain et al., 2002; Carpentier and Potter, 2007). The sound level was 88–90 dBA, exceeding background sound level.

### Measurement of Sound Environment

Previous studies indicate that acoustic perception of urban open spaces can be affected by sound pressure level (Yu and Kang, 2010; Xie et al., 2012). Since the measurement time for the current study was 9:00–11:00 a.m. and 14:00–16:00 p.m., its crowd density was less than 0.05/m^2^; thus, the influence of the number of people on the square on the acoustic environment could be ignored (Meng and Kang, 2015). Therefore, acoustic environmental measurement was carried out point by point, not simultaneously.

To measure the sound environment, the area was divided into 6 m × 6 m units (Li and Meng, 2015). The equivalent continuous A-weighted sound pressure level (*L*_Aeq_) was immediately recorded using an 801 sound-level meter after each observation was completed. During the measurement, the sound-level meter was adjusted to the slow speed (Kang and Zhang, 2010). Additionally, the distance between the measurement location and walls and other major reflective surfaces was ensured to be at least 1 m, and the distance between the measurement location and the ground was 1.2–1.5 m (Barron and Foulkes, 1994; Zahorik, 2002). One measurement was performed every 10 s. The data for each location were recorded for 5 min. A mean value was calculated to obtain the corresponding *L*_Aeq_ (Zhang et al., 2016).

**FIGURE 2:**
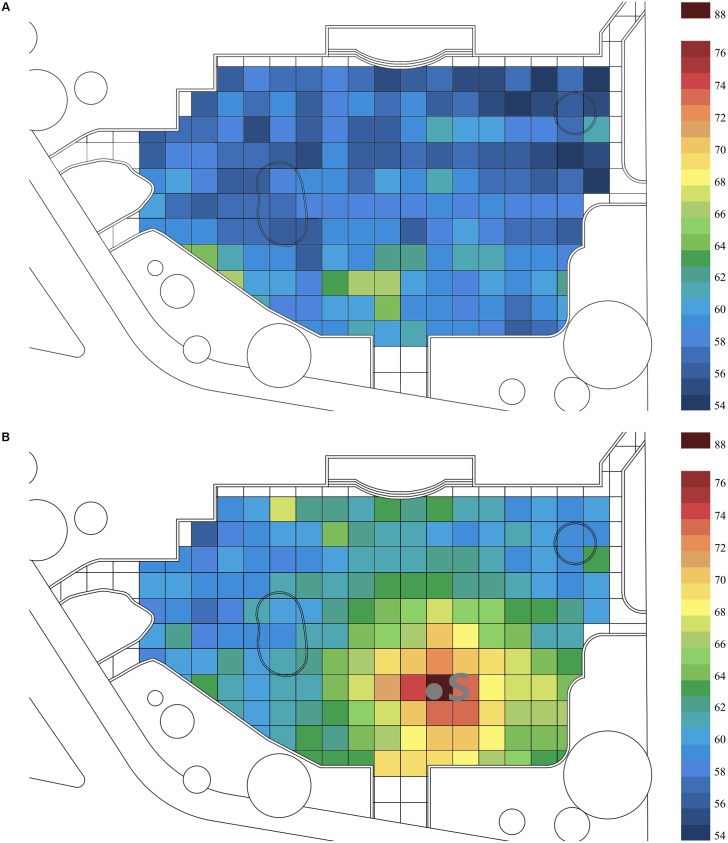
The distribution of sound pressure level in the case site (S means sound source): **(A)** with music and **(B)** without music.

The sound field in the urban open space can be seen in **Figure 2** (S means sound source). When there was no music sound source in the square, the background sound pressure level was 56.7 dB. When music was present, the sound pressure level at 2 m away from the music sound source was 88.3, or 31.6 dB higher than that without music. With the effect of musical sound source considered, the equivalent A-weighted sound pressure level reduced constantly with increasing distance in the square. From 2 to 12 m away from the sound source, the sound pressure level reduced at 18.3 dB; from 12 to 24 m away from the sound source, the sound pressure level reduced at 6.4 dB; and from 24 to 36 m away from the sound source, the sound pressure level reduced at 3.4 dB. The attenuation degree of the equivalent A-weighted sound level was mainly determined by the degree of enclosure for the space.

### Observation of Crowd Behaviors

In previous soundscape research, the investigation methods were classified as questionnaires and observations (Meng and Kang, 2016; Meng et al., 2017a). The questionnaires mainly focused on subjective evaluation indexes such as sound comfort, subjective loudness, and sound preference. This study involves measurements of crowd movement and non-movement behaviors, including path, speed, location of the stop points, etc., which is difficult to assess by questionnaire interview; thus, observation was the method used. To avoid any biases in the observation process, this study used an unmanned aerial vehicle (UAV; Oakes and North, 2008); the UAV was flown at a height of 100 m, since at that height, people on the square could not hear the noise of the UAV (Sinibaldi and Marino, 2013). The observations were made under completely natural conditions, and since the subjects generally did not know that they were being observed, their behaviors were genuine; thus, the results were more reliable.

Each video shot by the UAV lasted 20–25 min. Videos of 10–15 groups for every situation were shot to ensure stochastic behavior in the measurement (Meng and Kang, 2013). Meanwhile, one photograph was taken every 10 s. The behaviors of the subjects were then classified and analyzed statistically, based on the review of videos and photos in the laboratory.

### Statistical Analysis of Crowd Behaviors

In this study, different samples were used for different behaviors, to capture the different times of collection of the behaviors. For instance, the period needed to collect two samples of passing by behaviors was the same as that for three samples of walking around behavior and of five samples for sitting behavior. In all, 51 samples were collected for passing by behavior: 26 samples (12 males and 14 females) without music and 25 (13 males and 12 females) with music; 84 samples were collected for walking around behavior, of which 43 samples (20 males and 23 females) were without music and 41 samples (20 males and 21 females) with music; and 123 samples were collected for sitting behavior, 63 without music and 60 with music. In preliminary study, it was found that proportions of males and females engaging in given behaviors at the case site are generally equal (Meng et al., 2017a). In order to use the *T*-test to compare the samples, therefore, 24 samples (12 males and 12 females) without music and 24 samples (12 males and 12 females) with music were randomly selected for passing by behavior, 40 samples (20 males and 20 females) with music and 40 samples (20 males and 20 females) without music for walking around behavior, and 60 samples without music and 60 with music for sitting behavior.

In the present study, the power analysis was used to test sample sizes (Carpentier and Potter, 2007). The results showed that the power of samples for passing by behavior is 0.60, *p* = 0.04 with effect size 0.6; for walking around behavior, power is 0.77, *p* = 0.03, with effect size 0.6; and for sitting behavior, power is 0.87, *p* = 0.01 with effect size 0.6. This indicates that all samples were sufficient.

#### The Path and Speed of Passing by Behaviors

**FIGURE 3:**
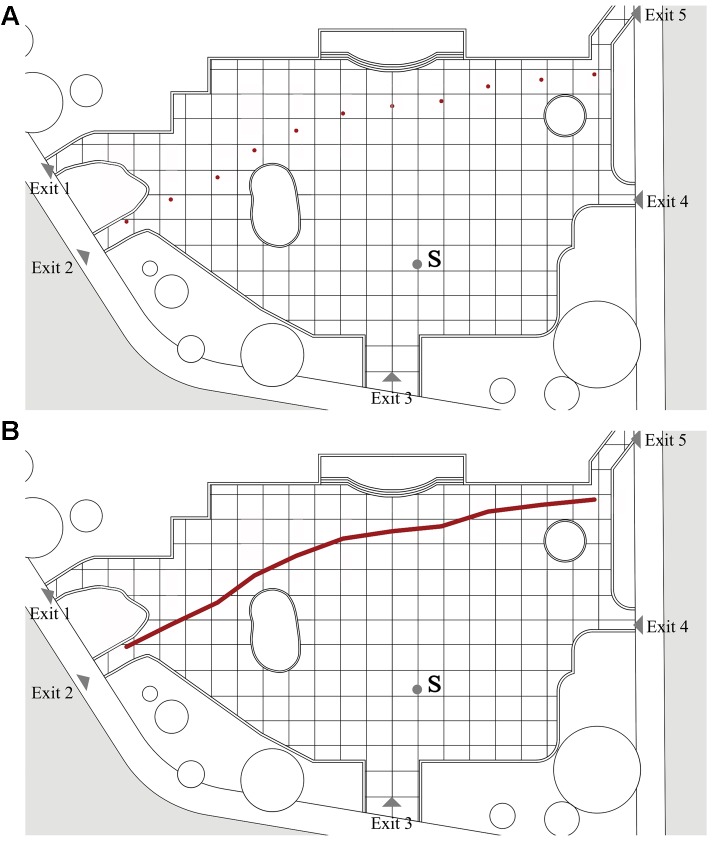
The calculation process of speed of passing by behavior: **(A)** path points of passing by behavior and **(B)** path of passing by behavior.

To study the effect of music on the path and speed of passing by behaviors in urban open spaces, 48 samples, including 24 without music and 24 with, were selected for observation from the videos shot by the UAV. The locations of the entrances and exits are shown in **Figure 3**. In previous studies, the path was represented by a set of dots, and each dot was considered a relatively independent process (Ye et al., 2012). The entire process of passing by behavior was thus viewed as a collection of data flows between the many dots. As revealed in **Figure 3A** using passing by behavior as an example, the path points were labeled with round dots representing the subject’s position as observed every 10 s in the photography taken by the UAV. Thus, as **Figure 3B** shows, the path was in turn conceived by connecting all of the points.

The calculation process of the mean speed was as follows (Marušić, 2011; Ye et al., 2012):

Vn = ΔLn/ΔTn

where Δ*Ln* is the distance of dot Cn and dot Cn+1, Δ*Tn* is the time of dot Cn and dot Cn+1, *Vn* is the mean speed of distance of dot Cn and dot Cn+1, and mean speed: Δ*V* = (*V*1 + *V*2 + *V*3…… + *Vn*)/*n*.

The mean speed was a superimposition of data from 24 samples (with or without music). The unit was m/s.

#### The Path and Speed of Walking Around Behaviors

To study the effect of music on the path and speed of walking around behaviors in urban open spaces, 80 samples, including 40 samples without music and 40 samples with music, were selected for observation from the videos shot by the UAV. Based on the observations, the paths of walking around behavior were classified into four types. The data from each walking behavior were calculated as the mean of five occurrences, since previous studies indicate that the error of the mean of more than or equal to five times could be ignored. The calculation process for mean speed of walking behavior was the same as with passing by behavior. The results were the superimposition of data from 10 samples for each kind of path. The units used included m^2^ (area), m (perimeter), and m/s (speed).

#### The Crowd Density of Sitting Behaviors

To study the effect of music on the crowd density of a non-movement behavior in urban open spaces, crowd location was measured using the same photography method. Using sitting behavior as an example, one photograph shot by the UAV was selected every 2 min (Westover, 1989; Meng and Kang, 2015). In the laboratory, the locations of the crowd in the picture were labeled with round dots, and a 6 m × 6 m grid was used. The value obtained was divided by the measurement area to determine a mean value of crowd density as the average number of persons per square meter. The unit used for measurement was persons/m^2^. A total of 60 samples with music and 60 samples without music were used. The unit used for measurement was persons/m^2^ (Meng et al., 2017b).

## Results

### Effects of Music on Movement Behavior: Passing by Behavior

#### Path

**FIGURE 4:**
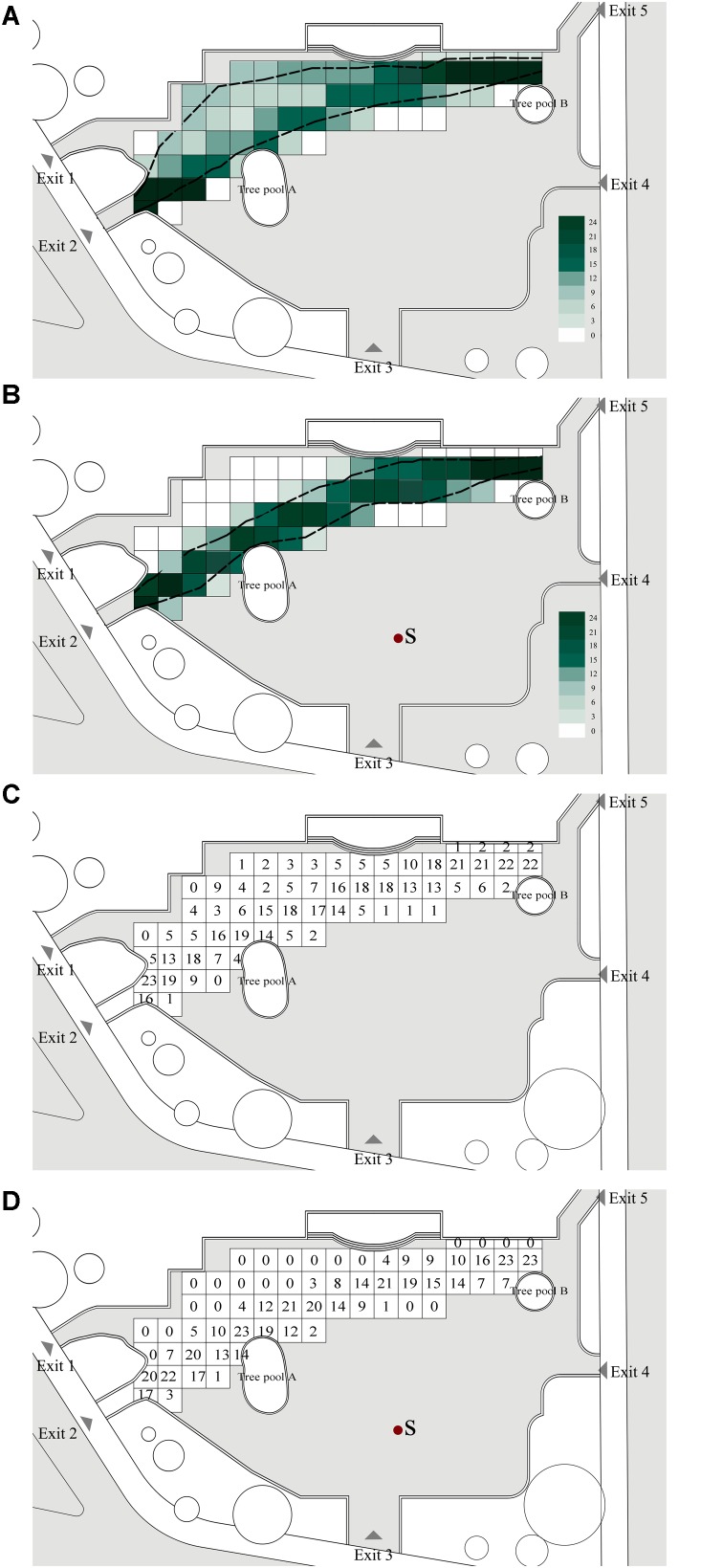
Effect of music on path of passing by behavior in the case site: **(A)** path without music, **(B)** path with music, **(C)** number of persons per grid without music, and **(D)** number of persons per grid with music. The dotted line indicates the boundary of passing by behaviors. The squares with different colors mean the numbers of passing by users, from 0 to 24 persons.

This section addresses the effects of music on the path of passing by behavior, which is shown in **Figure 4**, both with and without background music; the squares with different colors in **Figures 4A,B** indicate the numbers of users passing by, from 0 to 24 persons.

##### Without background music

As **Figure 4A** shows, 79.2% of people with passing by behavior selected a relatively short walking path. One possible reason for this is that when moving with a clear goal, passers-by often tended to choose the shortest path. This is usually a straight line approximately toward the goal, unless there is an obstacle (Gehl, 1987; Chen, 2009). It was found that 20.8% of people engaging in passing by behavior selected a relatively longer walking path, and even that 8.3% of them walked near the edge of the square. The areas covered by passing by behaviors were approximately 1436 m^2^.

##### With background music

As **Figure 4B** shows, 0% of people with passing by behavior walked near the edge of the square, and 87.5% of them selected the relatively shorter path to walk; 12.5% of people with passing by behavior passed by the square while walking close to the sound source. The areas covered by passing by behaviors were approximately 876 m^2^.

The area of passing by behavior, both with and without background music, is marked in **Figure 4C**, for the number of persons per grid without music, and **Figure 4D**, with music. Comparing these two group of numbers, it can be seen that the path boundaries, with and without background music, were generally significantly different, with independent-samples *T*-test *t* = 0.848, *p* = 0.018, and effect size = 0.412. The number of observations of people with passing by behaviors with a relatively short path close to the music sound source in the square was 8.3% higher with background music than without. Passing by behaviors near the edge of the square with background music were zero when compared to the square without background music. Passing by behaviors closer to the music sound with background music were 12.5% higher when compared to the square without background music. The areas covered by passing by behaviors can also be reduced with music. This means that the presence of music caused people to be more centralized and walk closer to the sound source when passing by.

#### Speed

In terms of the speed of the passing by behavior, the square was considered both with and without background music.

##### Without background music

The mean speed of the walking around behavior in the square was 1.30 m/s. The minimum speed was 1.09 m/s, and the maximum speed was 1.57 m/s.

##### With background music

The mean speed of the walking around behavior in the square was 1.30 m/s. The minimum speed was 1.06 m/s, and the maximum speed was 1.59 m/s.

The mean speed of the passing by behavior in the square with music was generally not significantly different from that of without music, with independent-samples *T*-test *t* = -0.208, *p* = 0.836, and effect size = 0.032.

Furthermore, exploring gender effects indicated that the path and speed of the passing by behavior for males and females, with and without background music, were generally not significantly different, with *T*-test *t* = 0.132, *p* = 0.732, and effect size = 0.051.

### Effects of Music on Movement Behavior: Walking Around Behavior

#### Path

**FIGURE 5:**
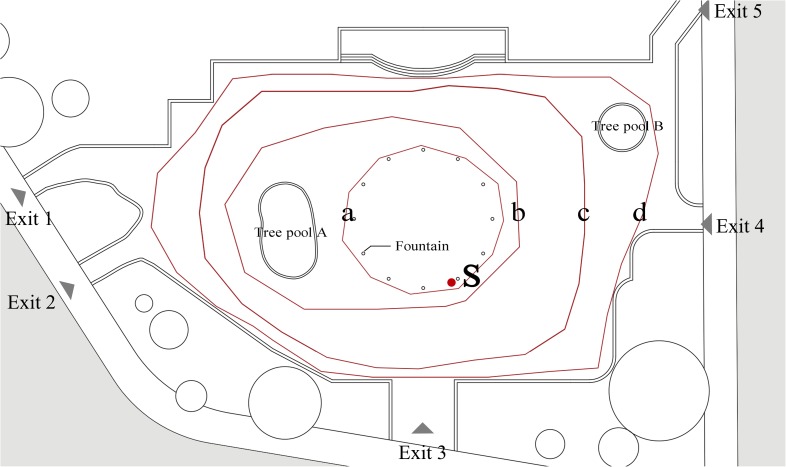
Four kinds of paths of walking around behavior.

This section addresses the effects of music on the path of walking around behavior. Previous studies indicate that the paths from movement behavior are not random, but rather they are regular and directional. In this case, the users’ paths in the square were influenced by environmental factors. Thus, based on the observations, the paths of walking around behavior were classified into four categories according to the location of boundaries and the water feature fence in the square. As **Figure 5** shows, path “a” represents walking around the fountain; path “b” implies walking around the fountain and tree pool A; path “c” represents walking around the boundary of the square except tree pool B; and path “d” implies walking around the boundary of the square including tree pool B. There were significant differences between the four paths, and therefore they were separated for a comparative analysis in which the square with and without background music was considered.

##### Without background music

**FIGURE 6:**
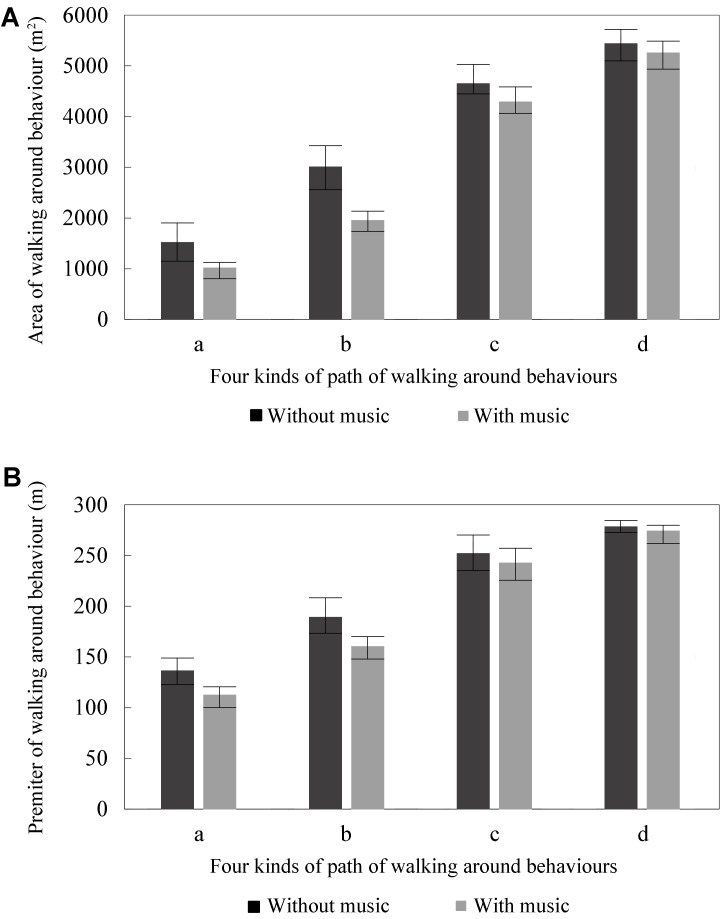
Effect of music on path of walking around behavior with and without music in the case site: **(A)** area and **(B)** perimeter.

As **Figure 6A** shows, the mean areas of the paths from walking around behavior in the square were 1535 (a), 3024 (b), 4668 (c), and 5259 m^2^ (d). As **Figure 6B** shows, the mean perimeters of the paths from walking around behavior in the square was 137 (a), 181 (b), 252 (c), and 274 m (d).

##### With background music

As **Figure 6A** shows, the mean areas of the paths from walking around behavior in the square were 1038 (a), 1973 (b), 4311 (c), and 5338 m^2^ (d). As **Figure 6B** shows, the mean perimeters of the paths from walking around behavior in the square were 113 (a), 160 (b), 243 (c), and 278 m (d).

The results indicated that the mean areas of walking around behavior in the square with background music were 32.36 (a), 30.74 (b), 7.66 (c), and 4.30% (d) smaller than that of the square without background music. Comparing the cases with and without background music indicated that the mean perimeters of walking around behavior in the square with background music were 17.34 (a), 15.14 (b), 3.68 (c), and 1.54% (d) smaller than that of the square without background music.

The ANOVA test was used to analysis the significance among music, category, and characteristics of walking around behavior, as shown in **Table 1**.

**Table 1:** ANOVA test for music, category, path and speed of walking around behavior.

Factor	Variables	Mean	*SD*	*F*	Sig.	Effect size
Path	A0	3621.9443	1524.38340	177.963	0.000	0.937
	A1	3165.3397	1794.34592	248.789	0.000	0.954
	P0	211.5420	57.27472	224.300	0.000	0.949
	P1	199.1993	67.67651	296.166	0.000	0.961
	S0	1.4300	0.16733	0.647	0.590	0.051
	S1	1.1403	0.17839	0.090	0.965	0.007
Music	Aa	1286.7890	350.52975	20.195	0.000	0.529
	Ab	2498.7895	700.22086	26.140	0.000	0.592
	Ac	4490.0245	567.88599	2.100	0.165	0.104
	Ad	5298.9650	245.73979	0.499	0.489	0.027
	Pa	125.2505	15.48015	29.500	0.000	0.621
	Pb	171.1615	17.66377	9.909	0.006	0.355
	Pc	248.2220	19.45561	1.158	0.296	0.060
	Pd	276.8485	8.06845	1.419	0.249	0.073
	Sa	1.2835	0.21607	8.339	0.010	0.317
	Sb	1.3075	0.25367	21.037	0.000	0.539
	Sc	1.2620	0.20776	13.300	0.002	0.425
	Sd	1.2875	0.23637	12.503	0.002	0.410

*A, area; P, perimeter, S, speed; a, b, c, and d, four categories; 0, without music; 1, with music.*

Compared with walking around behavior, with music and without, there were significant differences in areas at categories a and b, with ANOVA *p* = 0.000 and effect size = 0.592 (a) and 0.529 (b); and there were no significant differences in areas at categories c and d, with ANOVA *p* = 0.165 (c) and 0.489 (d) and effect size = 0.104 (c) and 0.027 (d). Similarly, there were significant differences in perimeters at categories a and b, with ANOVA *p* = 0.000 (a) and 0.006 (b), and effect size = 0.621 (a) and 0.355 (b), and no significant differences in perimeters at categories c and d, with ANOVA *p* = 0.296 (c) and 0.249 (d), and effect size = 0.060 (c) and 0.073 (d). A possible reason for these results in categories a and b is that the crowd may have tended to move toward sound stimuli and then walk around at a shorter distance away from the music; this is then similar to the results found for passing by behavior. Compared with categories a and b, a possible reason for the results in categories c and d is that the crowd at categories c and d was relatively far away from the music sound source and therefore the effect of music was not significant in these situations.

#### Speed

**FIGURE 7:**
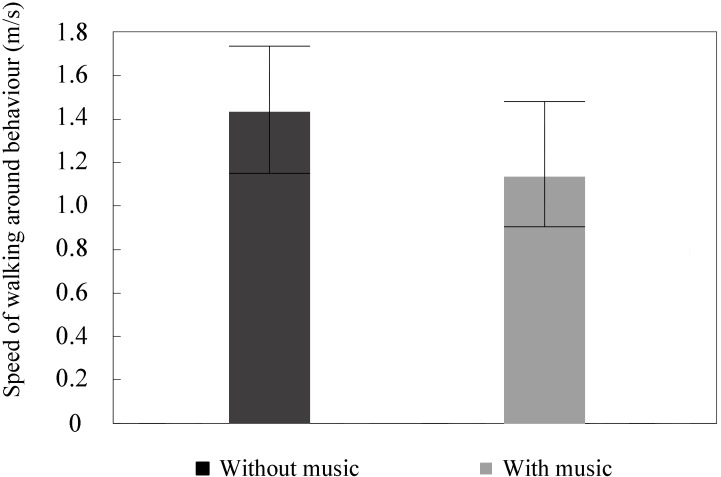
Effect of music on speed of walking around behavior with and without music in the square.

The mean speed of the four paths was analyzed first. The maximum difference of mean speeds among the four paths was 0.26 m/s without background music and 0.19 m/s with background music. It can be seen, from **Table 1**, that the speed of the paths in walking around behavior with background music was significantly slower than without background music in the four categories, with ANOVA *p* = 0.010 (a), 0.000 (b), 0.002 (c), and 0.002 (d), and effect size = 0.317 (a), 0.539 (b), 0.425 (c), and 0.410 (d). There were no significant differences between the four categories, with ANOVA *p* = 0.590 (without music) and 0.965 (with music), and effect size = 0.051 (without music) and 0.007 (with music). Therefore, the paths were merged to analyze the speed of walking around behavior. **Figure 7** shows the speed of walking around behavior in squares with and without background music.

##### Without background music

The mean speed of the paths for walking around behavior in the square was 1.43 m/s. The minimum speed was 1.15 m/s, and the maximum speed was 1.74 m/s.

##### With background music

The mean speed of the paths for walking around behavior in the square was 1.14 m/s. The minimum speed was 0.93 m/s, and the maximum speed was 1.49 m/s.

Furthermore, exploring gender effects indicated that the path and speed of walking around behavior for males and females, with and without background music, was generally not significantly different with *T*-test *t* = 0.211, *p* = 0.932, and effect size = 0.005.

### Effects of Music on Non-movement Behavior: Sitting Behavior

**FIGURE 8:**
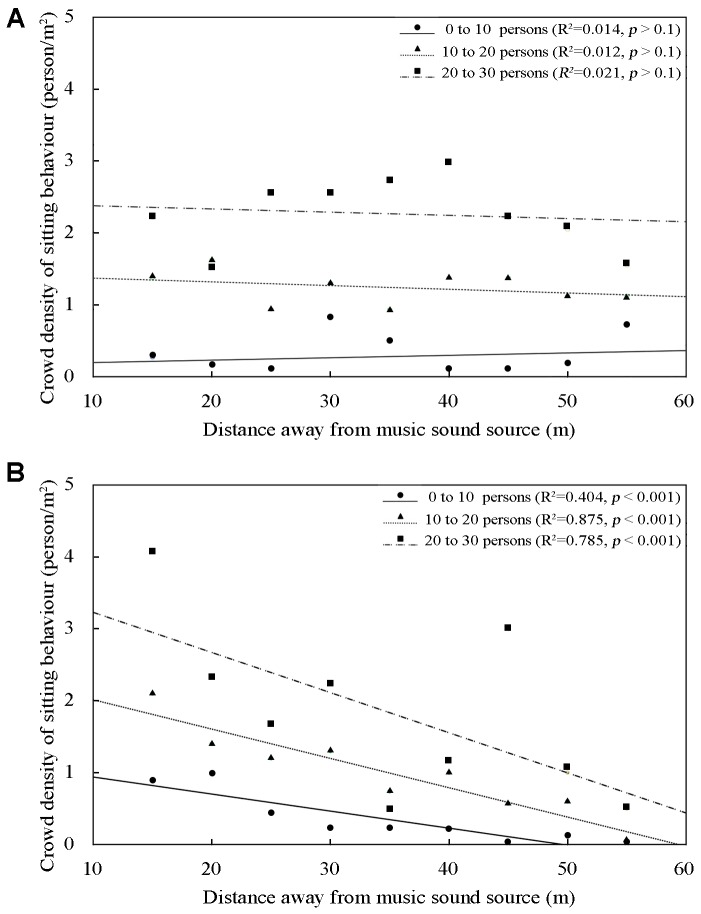
Relationship between crowd density of those with sitting behavior and distances away from music sound source in the square: **(A)** with music and **(B)** without music, where the solid line means 0–10 persons, the dotted line means 11–20 persons, and the chain line means 21–30 persons.

This section addresses the effects of music of sitting behavior on crowd density. According to the statistical analyses, the number of those exhibiting sitting behavior ranged from 0 to 30. The relationship between crowd density and distance away from the music sound source in the square is shown in **Figure 8**, where the solid line means 0–10 persons, the dotted line means 11–20 persons, and the chain line means 21–30 persons, along with the linear regression and the coefficient of determination *R*^2^. Results for observations with and without background music are discussed.

#### Without Background Music

As **Figure 8A** shows, there were no significant differences in sitting behavior by distance away from the music sound source, with linear regression *R*^2^ of 0.014 (0–10 persons), 0.012 (11–20 persons), and 0.021 (21–30 persons) and *p* > 0.1. The results indicated that sitting behavior remained randomly distributed over the case site with the increase of crowd density, and was generally not changed with different distance of sound sources. When the number of persons engaged in sitting behavior ranged from 0 to 10, 11 to 20, and 21 to 30, the crowd densities were respectively about 0.46, 1.27, and 1.96 persons/m^2^ within 15–20 m of the music sound source, and generally the same at 25–30 and 35–40 m.

#### With Background Music

It can be seen that sitting behavior increased with decreasing distance away from the music sound source, with linear regression *R*^2^ of 0.404 (0–10 persons), 0.875 (11–20 persons), and 0.785 (21–30 persons) and *p* < 0.001. It can be seen from **Figure 8B** that the crowd of persons engaged in sitting behavior decreased with increasing distance of music sound source. When the number of those exhibiting sitting behavior ranged from 0 to 10, the crowd densities were 0.89 persons/m^2^ within 15–20 m of the music sound source, 0.56 persons/m^2^ within 25–30 m, and 0.41 persons/m^2^ within 35–40 m. When the number of those exhibiting sitting behavior ranged from 10 to 20, the crowd densities were about 1.82 persons/m^2^ within 15–20 m of the music sound source. When the number of those exhibiting sitting behavior ranged from 20 to 30, the crowd densities were about 2.95 persons/m^2^ within 15–20 m of the music sound source. One possible reason for these results is that the frequency of the music heard is reduced as the distance from music sound source is increased. It is interesting to note that when the numbers exhibiting increase in sitting behavior, the inclination of the three corresponding linear trend curves fell faster. For example, when the number of those with sitting behavior ranged from 0 to 10, crowd density in the square with background music was reduced by 0.12 persons/m^2^ for every 5 m away from the source; when that number ranged from 20 to 30, crowd density was reduced by 0.28 person/m^2^ every 5 m.

The comparison reveals that the crowd density of those exhibiting sitting behavior in the square with background music was higher than that without background music, when the distance to sound source was relatively shorter, while the crowd density of sitting behavior in the square with background music was lower than that without background music, when the distance was relatively long. For example, when the number of those with sitting behaviors ranged from 20 to 30, at 15–20 m away from the music sound source, crowd density was 0.99 persons/m^2^ higher with background music than without, while at 35–40 m away from the music sound source, crowd density of those with sitting behaviors with background music was 0.99 persons/m^2^ lower than without.

## Discussion

The purpose of the present study was to explore the effect of music on movement behaviors, such as passing by behavior and walking around behavior, and non-movement behaviors, such as sitting behavior, in urban open spaces.

Regarding the effect of music on passing by behavior, as discussed in Section “Effects of Music on Movement Behavior: Passing by Behavior,” the speed of passing by behavior was generally not significantly affected by music, while the path of passing by behavior shifted closer to the music sound source. This is in contrast to Lavia et al. (2016), who found that when music was deployed, people’s walking speed through the other open spaces was slower. One possible reason for this is that the aims of passing by behavior are different in the two studies. In Lavia et al.’s (2016) study, the users are just intending to walk in the street. Some previous studies have pointed that when walkers do not have a clear purpose, their speed can be changed by landscape or environmental factors (Chen, 2009; Jia, 2012; Xie et al., 2013). In contrast, in the present study, the users are passing to go to work or school, and thus have a clear purpose, making it reasonable that the speed of their behavior was not affected by the background music. An investigation among students also pointed out that visual differences do not change the speed of going to school (Fiegel et al., 2014). As for the effect of sound on path of passing by behavior, some previous studies have indicated that some animals and people will change their path to be far away from traffic noise (Lambert et al., 1984; Lengagne, 2008), whereas in the present study, it can be seen that the path of the crowd can be changed to be nearer the music. These results reinforce that behaviors can be effectively changed using the urban soundscape (Husain et al., 2002; Kang and Zhang, 2010).

Regarding the effect of music on walking around behavior, as discussed in Section “Effects of Music on Movement Behavior: Walking Around Behavior,” the area, perimeter, and speed of the walking around behavior decrease with music. This result was the same as another study in which crowd behavior tended to move toward music (Jia, 2012). It is also interesting to note that a third study found that the children have different play behaviors with increasing distance from music (Holmes and Willoughby, 2005). In addition, the difference by the presence or absence of background music decreased as the area and perimeter increased. This was different from the effect of music on speed of passing by behavior, as the mean speed during walking around behavior with background music in the square was 0.29 m/s slower than without background music. These results show once more that behaviors without aims can be changed by environmental and landscape factors (Chen, 2009; Jia, 2012; Xie et al., 2013). Therefore, it can be concluded that the music drew people closer to the sound source and slowed their speed of walking. These results were the same as those found by Lavia et al. (2016) for other urban open spaces. A possible reason for this is that when hearing background music, users feel more comfortable; thus, their speed of walking around slows. Another reason may be that the presence of background music improves the feeling of safety in the environment; thus, background music contributes to building an urban slow space, which is more suitable for residents’ health (Ye et al., 2012).

Third, regarding the effect of music on the crowd density of those exhibiting sitting behavior, as discussed in Section “Effects of Music on Non-movement Behavior: Sitting Behavior,” when there was no music, there was no significant difference in density no matter how close to the sound source they were located. However, as the distance from the sound source increased, crowd density of those with sitting behavior decreased accordingly. Some previous studies have pointed that when there is no music, there is no significant difference in the crowd density of those with sitting behavior in indoor spaces such as railway stations and underground shopping streets (Debrezion et al., 2009; Meng et al., 2013). In urban open spaces, Meng and Kang (2016) also found that human sound-related activities generally have little effect on the sitting behaviors of pedestrians. On the effect of music, this result proves once more the finding that music-related activities increased the number of persons who passed by who stood and watched (Meng and Kang, 2016). As with music, users can also be attracted to a location by some nature sounds, such as sounds of bird or water (Liu et al., 2013). Some previous studies have also pointed that the acoustic perception of music is usually more salient than that of nature sounds (Aletta et al., 2016); this may lead to the different changes in sitting behaviors.

There are a number of possible implications for the applied value of the present study. Certain soundscapes, such as some music, may lead pedestrians to different paths in urban open spaces; it will be useful in landscape design to further investigate ways to lead walkers to suitable paths in gardens, for instance. Moreover, in leisure spaces such as parks, music can be used to decrease the speed of users and help them enjoy the landscape carefully. Furthermore, in rest areas in squares, a public-address system can be used to broadcast music to increase non-movement behavior, which can effectively increase interactions of citizens.

As demonstrated in the literature review, there are many classifications of musical sounds; however, only typical musical sounds were used in this study. In future studies, different tempos, genres, contexts, and levels of familiarity of musical sounds could also be investigated for comparison. Also, the location of the sound source was fixed in the present study; it can be seen from other studies that different locations of sound sources may lead to varying acoustic perceptions (Kang and Zhang, 2010). Therefore, in future studies by the present authors, different locations of music sound sources will be designed to find out their different effects on behaviors. Regarding movement and non-movement behaviors, only their speed and path were investigated in this work, whereas some previous studies have also pointed that characteristics of these behaviors such as duration and location also have effects that will be important for landscape design and urban planning (Lepore et al., 2016); therefore, in future studies, the present authors will further explore and explain these factors also.

## Author Contributions

All authors carried out the study, designed the experiments, and wrote and critically reviewed the paper. QM and JK carried out the experiments. TZ analyzed the results.

## Conflict of Interest Statement

The authors declare that the research was conducted in the absence of any commercial or financial relationships that could be construed as a potential conflict of interest.

## References

[B1] Aletta F. Lepore F. Kostara-Konstantinou E. Kang J. Astolfi A. (2016). An experimental study on the influence of soundscapes on people’s behaviour in an open public space. *Appl. Sci.* 6:276 10.3390/app6100276

[B2] Ashihara Y. (1985). *External Space Design.* Beijing: China Architecture & Building Press.

[B3] Barron M. Foulkes T. J. (1994). Auditorium acoustics and architectural design. *J. Acoust. Soc. Am.* 96:612 10.1121/1.410457

[B4] Brander L. M. Koetse M. J. (2011). The value of urban open space: meta-analyses of contingent valuation and hedonic pricing results. *J. Environ. Manage.* 92 2763–2773. 10.1016/j.jenvman.2011.06.019 21763064

[B5] Carpentier F. R. D. Potter R. F. (2007). Effects of music on physiological arousal: explorations into tempo and genre. *Media Psychol.* 10 339–363. 10.1080/15213260701533045

[B6] Chen S. (2009). *Study on Behaviour Vitality of in City Square.* Master’s thesis, Central South University, Changsha.

[B7] Dai Y. (2014). *Study on User Behaviour in an Urban Square Space.* Master’s thesis, Central South University, Changsha.

[B8] Debrezion G. Pels E. Rietveld P. (2009). Modelling the joint access mode and railway station choice. *Transp. Res. Part E* 45 270–283. 10.1016/j.tre.2008.07.001

[B9] dela Fuente de Val G. Atauri J. A. Lucio J. V. (2006). Relationship between landscape visual attributes and spatial pattern indices: a test study in Mediterranean-climate landscapes. *Landsc. Urban Plan.* 77 393–407. 10.1016/j.landurbplan.2005.05.003

[B10] Fiegel A. Meullenet J. F. Harrington R. J. Humble R. Seo H. S. (2014). Background music genre can modulate flavor pleasantness and overall impression of food stimuli. *Appetite* 76 144–152. 10.1016/j.appet.2014.01.079 24530691

[B11] Floyd M. F. (1998). Getting beyond marginality and ethnicity: the challenge for race and ethnic studies in Leisure Research. *J. Leis. Res.* 30 3–22. 10.1080/00222216.1998.11949816

[B12] Franěk M. van Noorden L. Režný L. (2014). Tempo and walking speed with music in the urban context. *Front. Psychol.* 5:1361. 10.3389/fpsyg.2014.01361 25520682 PMC4251309

[B13] Gehl J. (1987). *Life between Buildings: Using Public Space.* New York, NY: Van Nostrand Reinhold Company, Inc.

[B14] Gomez P. Danuser B. (2004). Affective and physiological responses to environmental noises and music. *Int. J. Psychophysiol.* 53 91–103. 10.1016/j.ijpsycho.2004.02.002 15210287

[B15] Guéguen N. Jacob C. Guellec H. L. Morineau T. Lourel M. (2008). Sound level of environmental music and drinking behaviour: a field experiment with beer drinkers. *Alcohol. Clin. Exp. Res.* 32 1795–1798. 10.1111/j.1530-0277.2008.00764.x 18647281

[B16] Holmes E. Willoughby T. (2005). Play behaviour of children with autism spectrum disorders. *J. Intellect. Dev. Disabil.* 30 156–164. 10.1080/13668250500204034

[B17] Husain G. Thompson W. F. Schellenberg E. G. (2002). Effects of musical tempo and mode on arousal, mood, and spatial abilities. *Music Percept.* 20 151–171. 10.1525/mp.2002.20.2.151

[B18] Hwang R. L. Lin T. P. Matzarakis A. (2011). Seasonal effects of urban street shading on long-term outdoor thermal comfort. *Build. Environ.* 46 863–870. 10.1016/j.buildenv.2010.10.017

[B19] ISO 12913-1 (2014). *Acoustics- Soundscape-Part1: Definition and Conceptual Framework.* Geneva: International Organisation for Standardization.

[B20] Jia S. Q. (2012). *Crowd Behaviour in Soundscape.* Master’s thesis, Harbin Institute of Technology, Harbin.

[B21] Kang J. (2017) From dBA to soundscape indices: managing our sound environment. *Front. Eng. Manag.* 4 184–192. 10.15302/J-FEM-2017026

[B22] Kang J. Zhang M. (2010). Semantic differential analysis of the soundscape in urban open public spaces. *Build. Environ.* 45 150–157. 10.1016/j.buildenv.2009.05.014

[B23] Lambert J. Simonnet F. Vallet M. (1984). Patterns of behaviour in dwellings exposed to road traffic noise. *J. Sound Vib.* 92 159–172. 10.1016/0022-460X(84)90553-4

[B24] Lavia L. Witchel H. J. Kang J. Aletta F. (2016). “A preliminary soundscape management model for added sound in public spaces to discourage anti-social and support pro-social effects on public behaviour,” in *Proceedings of DAGA Conference*, (Aachen), 1–4. 10.13140/RG.2.1.1392.4242

[B25] Lengagne T. (2008). Traffic noise affects communication behaviour in a breeding anuran. *Hyla arborea*. *Biol. Conserv.* 141 2023–2031. 10.1016/j.biocon.2008.05.017

[B26] Lepore F. Kostara-Konstantinou E. Aletta F. Astolfi A. Kang J. (2016). “A preliminary investigation about the influence of soundscapes on people’s behaviour in an open public space,” in *Proceedings of Internoise*, (Hong Kong), 5219–5224.

[B27] Lewin K. Heider F. Heider G. M. (1936). *Principles of Topological Psychology.* New York, NY: McGraw-Hill 10.1037/10019-000

[B28] Li J. N. Meng Q. (2015). Study on the soundscape in commercial pedestrian streets. *Tech. Acoust.* 34 326–329.

[B29] Liu J. Kang J. Luo T. Behm H. Coppack T. (2013). Spatiotemporal variability of soundscapes in a multiple functional urban area. *Landsc. Urban Plan.* 115 1–9. 10.1016/j.landurbplan.2013.03.008

[B30] Marcus C. C. Francis C. (1998). *People Places: Design Guidelines for Urban Open Space.* New York, NY: John Wiley & Sons, Inc.

[B31] Marušić B. G. (2011). Analysis of patterns of spatial occupancy in urban open space using behaviour maps and GIS. *Urban Des. Int.* 16 36–50. 10.1057/udi.2010.20

[B32] Meng Q. Kang J. (2013). Influence of social and behavioural characteristics of users on their evaluation of subjective loudness and acoustic comfort in shopping malls. *PLoS One* 8:e54497. 10.1371/journal.pone.0054497 23336003 PMC3545882

[B33] Meng Q. Kang J. (2015). The influence of crowd density on the sound environment of commercial pedestrian streets. *Sci. Total Environ.* 511 249–258. 10.1016/j.scitotenv.2014.12.060 25546463

[B34] Meng Q. Kang J. (2016). Effect of sound-related activities on human behaviours and acoustic comfort in urban open spaces. *Sci. Total Environ.* 573 481–493. 10.1016/j.scitotenv.2016.08.130 27572540

[B35] Meng Q. Kang J. Jin H. (2013). Field study on the influence of spatial and environmental characteristics on the evaluation of subjective loudness and acoustic comfort in underground shopping streets. *Appl. Acoust.* 74 1001–1009. 10.1016/j.apacoust.2013.02.003

[B36] Meng Q. Sun Y. Kang J. (2017a). Effect of temporary open-air markets on the sound environment and acoustic perception based on the crowd density characteristics. *Sci. Total Environ.* 60 1488–1495. 10.1016/j.scitotenv.2017.06.017 28605866

[B37] Meng Q. Zhang S. Kang J. (2017b). Effects of typical dining styles on conversation behaviours and acoustic perception in restaurants in China. *Build. Environ.* 121 148–157. 10.1016/j.buildenv.2017.05.025

[B38] Milliman R. E. (1982). Using background music to affect the behavior of supermarket shoppers. *J. Mark.* 46 86–91. 10.2307/1251706

[B39] Myers M. (1975). Decision making in allocating metropolitan open space: state of the art. *Trans. Kansas Acad. Sci.* 78 149–153. 10.2307/3627339

[B40] Natarajan K. Rajendra A. U. Alias F. Tiboleng T. Puthusserypady S. K. (2004). Nonlinear analysis of EEG signals at different mental states. *Biomed. Eng. Online* 3:7. 10.1186/1475-925X-3-7 15023233 PMC400247

[B41] North A. C. Hargreaves D. J. Hargreaves J. J. (2004). Uses of music in everyday life. *Music Percept.* 22 41–77. 10.1525/mp.2004.22.1.41

[B42] Oakes S. North A. C. (2008). Reviewing congruity effects in the service environment musicscape. *Int. J. Serv. Ind. Manage.* 19 63–82. 10.1108/09564230810855716

[B43] Orr T. J. Myles B. S. Carlson J. K. (1998). The impact of rhythmic entrainment on a person with autism. *Focus Autism Other Dev. Disabl.* 13 163–166. 10.1177/108835769801300304

[B44] Payne L. L. Mowen A. J. Orsega-Smith E. (2002). An examination of park preferences and behaviors among urban residents: the role of residential location, race, and age. *Leis. Sci.* 24 181–198. 10.1080/01490400252900149

[B45] Ren X. Kang J. (2015). Interactions between landscape elements and tranquility evaluation based on eye tracking experiments. *J. Acoust. Soc. Am.* 5 3019–3022. 10.1121/1.4934955 26627775

[B46] Sakharov D. S. Davydov V. I. Pavlygina R. A. (2005). Intercentral relations of the human EEG during listening to music. *Hum. Physiol.* 31 27–32. 10.1007/s10747-005-0065-5 16122031

[B47] Sinibaldi G. Marino L. (2013). Experimental analysis on the noise of propellers for small UAV. *Appl. Acoust.* 74 79–88. 10.1016/j.apacoust.2012.06.011

[B48] Styns F. Van N. L. Moelants D. Leman M. (2007). Walking on music. *Hum. Mov. Sci.* 26 769–785. 10.1016/j.humov.2007.07.007 17910985

[B49] Thompson C. W. (2002). Urban open space in the 21st century. *Landsc. Urban Plan.* 60 59–72. 10.1016/S0169-2046(02)00059-2

[B50] Thwaites K. Helleur E. Simkins I. M. (2005). Restorative urban open space: exploring the spatial configuration of human emotional fulfilment in urban open space. *Landsc. Res.* 30 525–547. 10.1080/01426390500273346

[B51] Wang C. (2014). *Simulation Research on Occupant Energy-Related Behaviours in Building.* Doctoral dissertation, Tsinghua University, Beijing.

[B52] Westover T. N. (1989). Perceived crowding in recreational settings an environment-behavior model. *Environ. Behav.* 21 258–276. 10.1177/0013916589213002

[B53] Whitlock S. (2004). The market and the city: square, street, and architecture in early modern Europe. *J. Mod. Hist.* 79 649–651. 10.1086/523218

[B54] Xie H. Kang J. Mills G. H. (2012). Sound power levels of typical medical equipment in intensive care units. *Acta Acust. United Ac.* 98 659–666. 10.3813/AAA.918545

[B55] Xie H. Kang J. Mills G. H. (2013). Behaviour observation of major noise sources in critical care wards. *J. Crit. Care* 28 1109.e5–1109.e18. 10.1016/j.jcrc.2013.06.006 23927941

[B56] Ye P. Wang H. Gao F. (2012). A preliminary study on the research method of urban public space environment behaviour based on GPS: a Case Study of Shengli Square in Hefei. *Archit. J.* 2012 28–33.

[B57] Yu L. Kang J. (2010). Factors influencing the sound preference in urban open spaces. *Appl. Acoust.* 71 622–633. 10.1016/j.apacoust.2010.02.005

[B58] Yuan W. Tan K. H. (2011). A model for simulation of crowd behaviour in the evacuation from a smoke-filled compartment. *Phys. A* 390 4210–4218. 10.1016/j.physa.2011.07.044

[B59] Zahorik P. (2002). Assessing auditory distance perception using virtual acoustics. *J. Acoust. Soc. Am.* 111 1832–1846. 10.1121/1.1458027 12002867

[B60] Zakariya K. Harun N. Z. Mansor M. (2014). Spatial characteristics of urban square and sociability: a review of the city square. *Melb. Proc. Soc. Behav. Sci.* 2014 678–688. 10.1016/j.sbspro.2014.10.099

[B61] Zhang D. Zhang M. Liu D. Kang J. (2016). Soundscape evaluation in Han Chinese Buddhist temples. *Appl. Acoust.* 111 188–197. 10.1016/j.apacoust.2016.04.020

[B62] Zhang Y. Yang Z. Yu X. (2006). Measurement and evaluation of interactions in complex urban ecosystem. *Ecol. Model.* 196 77–89. 10.1016/j.ecolmodel.2006.02.001

